# Effects of Robot-Assisted Gait Training in Individuals with Spinal Cord Injury: A Meta-analysis

**DOI:** 10.1155/2020/2102785

**Published:** 2020-03-21

**Authors:** Chia-Ying Fang, Jia-Ling Tsai, Guo-Sheng Li, Angela Shin-Yu Lien, Ya-Ju Chang

**Affiliations:** ^1^School of Physical Therapy and Graduate Institute of Rehabilitation Science, College of Medicine, Chang Gung University, No. 259, Wenhua 1st Rd., Guishan Dist., Taoyuan, Taiwan; ^2^School of Nursing, College of Medicine, Chang Gung University, No. 259, Wenhua 1st Rd., Guishan Dist., Taoyuan, Taiwan; ^3^Healthy Aging Research Center, Chang Gung University, No. 259, Wenhua 1st Rd., Guishan Dist., Taoyuan, Taiwan; ^4^Division of Endocrinology and Metabolism, Department of Internal Medicine, Chang Gung Memorial Hospital, Linkou, No. 5, Fuhsing Str., Guishan Dist., Taoyuan, Taiwan; ^5^Neuroscience Research Center, Chang Gung Memorial Hospital, Linkou, No. 5, Fuhsing Str., Guishan Dist., Taoyuan, Taiwan

## Abstract

**Background:**

To investigate the effects of robot-assisted gait training (RAGT) on spasticity and pain in people with spinal cord injury (SCI). *Material and methods*. Four electronic databases (PubMed, Scopus, Medline, and Cochrane Central Register of Controlled Trials) were searched for studies published up to November 2019. Only human trials and of English language were included. The searched studies were reviewed and extracted independently by two authors. Randomized controlled trials (RCTs) and non-RCTs were pooled separately for analyses. Primary outcome measures included spasticity assessed by Ashworth scale (AS) or modified Ashworth scale (MAS) and pain assessed by VAS. Secondary outcome measures included lower extremity motor score (LEMS) and walking ability (i.e., 6-minute walk test, 10-meter walk test).

**Results:**

A total of 225 studies were identified. Eighteen studies (7 RCTs and 11 non-RCTs) including 301 subjects met inclusion criteria. The outcome measure of spasticity significantly improved in favor of RAGT group in non-RCTs (AS: 95%CI = −0.202 to -0.068, *p* ≤ 0.001; MAS: 95%CI = −2.886 to -1.412, *p* ≤ 0.001). The results on pain did not show significant change after RAGT in either RCTs or non-RCTs. LEMS and walking ability significantly increased in favor of RAGT.

**Conclusions:**

RAGT can improve spasticity and walking ability in people with SCI. The probable reason for no significant change in pain after RAGT is floor effect. RAGT is beneficial for normalizing muscle tone and for improving lower extremity function in people with SCI without causing extra pain.

## 1. Introduction

Spinal cord injury (SCI) usually causes unreversible motor and sensory impairments. The incidence of SCI is 40 to 80 new cases per million people per year from all causes, depending on the country. For traumatic SCI, the ratio of male-to-female is around 2 : 1 [1]. SCI results in weak or paralyzed muscles, atrophy, walking disability, sensory dysfunction, and autonomic disorders such as autonomic dysreflexia [2]. Spasticity and pain are also some consequences of SCI affecting locomotor and quality of life [3].

The prevalence of spasticity after SCI is 65% at discharge from hospital [4]. In chronic stage, the prevalence is higher. Andresen et al. reported that, in chronic SCI, 71% of patients had spasticity, from the self-reported questionnaire [5]. Severe spasticity is not only detrimental to patients' walking and motor function [6] but is also related to the presence of pain, lower quality of life, and daily activities [7, 8]. Dipiro and colleagues reported that the self-reported frequency of medication usage on spasticity did not significantly decrease from baseline to 5 years of follow-up in chronic SCI [9]. Therefore, finding a treatment strategy that can decrease spasticity and the use of medication might be beneficial to people with SCI.

The prevalence of chronic pain is high in people with SCI. Previous studies reported that the prevalence was around 84% and 73% in Canada and Denmark, respectively [5, 10]. Musculoskeletal pain is the most common type of chronic pain and presents early following spinal cord injury [11, 12]. The proportion of patients feeling at-level neuropathic pain is higher than below-level neuropathic pain [12]. Pain is highly correlated with poor mood, self-perceived health [12], physical functioning [13], and low quality of life [5] in SCI.

Walking ability is one of the rehabilitation goals of people with SCI, especially in people with incomplete injury. To achieve functional walking, patients require not only appropriate muscle strength and nerve innervation but also proper endurance and less fatigue. Fatigue impacts on function in 57% of individuals with SCI [14]. It is also more prevalent among younger SCI and SCI with shorter duration of disability [15]. Clinically, there are several commonly used measurement tools, such as 6-minute walk test (6MWT), 10-meter walk test (10MWT), timed up and go (TUG), Walking Index for Spinal Cord Injury (WISCI), and Functional Independence Measure-Locomotion (FIM-L), each assesses different aspects of walking ability. For example, 10MWT and 6MWT have been shown to be valid and reliable to measure ambulatory ability for individuals with SCI [16], and 6MWT has been suggested to be a good assessment tool of endurance [16, 17]. TUG is a simple and quick test to assess a person's mobility and balance and correlates well with gait speed for frail elderly [18] and endurance in chronic stroke [19]. FIM-L and WISCI address on the need of assistance when performing functional tasks. FIM-L measures the functional status (walking/wheelchair and stairs) of a person based on the level of assistance he or she requires [20]; it can be considered as an evaluation of the gait ability in activities of daily living. WISCI scores the walking ability according to the need for physical assistance, braces, and walking aids [21]. Manual muscle testing (MMT) assesses lower extremity motor score (LEMS) according to American Spinal Injury Association (ASIA).

Rehabilitation for improving pain, spasticity, and walking ability is always a challenge for clinicians. The use of robot-assisted gait training (RAGT) in the field of rehabilitation has become more widespread since this training is not limited by the individuals' muscle paralysis level. Current systems of RAGT include Lokomat (Hocoma AG, Switzerland), G-EO systemTM (Reha Technology AG, Switzerland), Walkbot (P&S Mechanics Co., Ltd, Korea), and ReoAmbulatorTM (Motorika, USA Inc.) [22]. RAGT provides repetitive and functional task training which induces greater activation of the sensorimotor cortex (S1, S2) and cerebellar regions [23]. A meta-analysis revealed that RAGT improved walking endurance, walking independence, and lower limb muscle strength, but did not reduce spasticity [24]. Other than task-specific training, RAGT provides proprioceptive inputs to lower extremities. According to gate control theory, large fiber activation might be able to block noxious small fiber afferents which cause pain and spasticity. Previous studies revealed that sensorimotor activity by treadmill training decreased pain behavior and nociceptive fiber density in the spinal dorsal horn in acute, subchronic, and chronic SCI mice model [25, 26]. Previous studies also reported that rhythmic passive movement could induce spinal circuitry reorganization, restore postactivation depression, and decrease spasticity in patients with SCI [27]. Therefore, it is plausible to hypothesize that RAGT can reduce pain and spasticity. In the past, much work has been done on investigating the effect of RAGT on walking performance, but reports of its effect on pain and spasticity were rare.

The purpose of this meta-analysis was to compare the effects of RAGT on spasticity and pain with those of other treatments after SCI.

## 2. Methods

This review integrated the results from relevant studies by following the systematic review and meta-analysis guidelines outlined in the Preferred Reporting Items for Systematic Review and Meta-Analysis (PRISMA) statement [28].

### 2.1. Types of Participants

This study included only SCI subjects, regardless of traumatic or nontraumatic lesion, the time since injury, age, and sex.

### 2.2. Types of Interventions

Any kind of RAGT compared with other training modalities or no training for lower limbs was included.

### 2.3. Outcome Measures

Primary outcome measures were spasticity and pain. Spasticity was assessed by modified Ashworth scale (MAS) or Ashworth scale (AS) for lower limbs. Pain was assessed by the visual analog scale (VAS). The VAS is widely used to assess self-perceived pain [29]. It is a 10-centimeter line in which 0 represents no pain and 10 at the right edge means intolerable pain. Participants subjectively reported their pain condition on the VAS scale.

Secondary outcome measures were LEMS and walking ability assessed by 6MWT, 10MWT, TUG, WISCI, and FIM-L. LEMS assessed motor score for lower limbs according to ASIA standard. 6MWT measured the walking distance in 6 minutes. 10MWT assessed the walking speed measuring the time necessary to walk 10 meters. 6MWT and 10MWT were reliable and responsive tools in assessing walking ability in incomplete SCI [30]. TUG assessed the time that a subject took to rise from a chair, walk three meters, turn around, walk back to the chair, and sit down. WISCI measured improvements in ambulation in persons with SCI by evaluating the amount of physical assistance, braces, or devices required to walk 10 meters. WISCI I scored from 1 to 19 and WISCI II from 1 to 20 [31]. FIM was an 18-item assessment of physical, psychological, and social function. The assessor graded the functional status of a person based on the need of assistance [32].

### 2.4. Type of Studies

RCT, non-RCT, and crossover trials (only the RAGT period) were included in analysis.

### 2.5. Searching Criteria

The searching criteria were limited to human studies and English language.

### 2.6. Data Sources

Four electronic databases (PubMed, Scopus, Medline (Proquest), and Cochrane Central Register of Controlled Trials (CENTRAL)) before November 2019 using Medical Subject Heading terms combined with keywords, such as robotics, spinal cord injury, pain, and spasticity, were processed in title, abstract, and keywords. [Supplementary-material supplementary-material-1] shows the combinations used.

### 2.7. Study Selection

Two authors independently searched and screened the titles, abstracts, and literatures to identify potentially relevant studies. Then, full texts of relevant studies were obtained and assessed to determine whether the articles met the inclusion criteria. Any disagreement was discussed and solved with the third author to reach a consensus in every relevant detail.

### 2.8. Data Extraction and Management

Two authors extracted data independently from included studies and filled into an extraction form. The following data were extracted: (1) authors; (2) year of publication; (3) study design; (4) inclusion/exclusion criteria; (5) subject demographics (age, gender, number of subjects, level of lesion, classification of ASIA, duration of injury); (6) intervention; (7) outcome measures; and (8) summary of the results.

Data at baseline and at the end of the intervention were extracted for the analysis of the effect of training. Measurements during the intervention or at follow-up were excluded due to inconsistent measuring time points used across different studies. Studies were excluded if necessary outcome measures were missing or not measured.

### 2.9. Quality Assessment

The methodological quality of the selected RCTs was independently assessed by two authors using the Cochrane risk of bias assessment tool [33]. For the assessment of the methodological quality of the selected cohort studies and clinical trials, the Newcastle Ottawa Scale [34, 35] was employed and done by two authors independently. Any disagreement was resolved through discussion and consensus with the third author.

### 2.10. Statistical Analysis

RCTs and non-RCTs were grouped and analyzed separately. Statistical analysis was performed using Comprehensive Meta-Analysis (CMA) version 3 to analyze the treatment effect. Mean differences and 95% confidence interval (CI) were calculated for each primary and secondary outcome. Random effect models were used to calculate the pooled mean difference estimates if heterogeneity occurred. Fixed effects models were used to calculate the pooled mean difference estimates if no heterogeneity occurred.

## 3. Results

### 3.1. Studies Included

**Figure 1:**
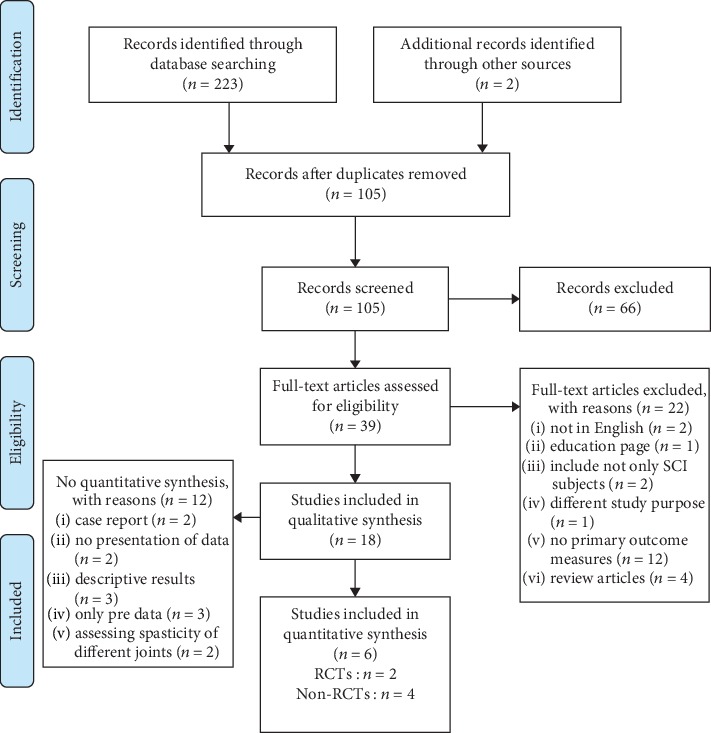
Flow diagram of the study selection process.

A total of 223 studies from electronic databases and two studies from the reference lists of included studies were identified. In these, 105 of the selected studies were duplicates and thus were removed from analysis. Out of the retained studies, 18 studies were retained for quality synthesis which included 7 RCTs and 11 non-RCTs. The flow of studies through the review process is shown in Figure 1. Six studies were included after review for quantitative synthesis of which the characteristics are shown in Table 1 and Table 2.

#### 3.1.1. Excluded Studies

After screening, a total of 66 studies were eliminated. The reasons for exclusion were as follows: texts not in English version, manuscripts in the form of education page, subjects of the studies included other diagnostic groups, study purpose, and outcome measures did not meet our inclusion criteria.

#### 3.1.2. Study Location

From the 18 studies, 7 trials were done in the United States [36–42], 2 in Spain [43, 44], 2 in Switzerland [45, 46], 2 in Canada [47, 48], 2 in Italy [49, 50], 2 in Japan [51, 52], and one in Germany [53].

#### 3.1.3. Study Participants

A total of 222 participants from 7 RCTs were included. Seventy-nine participants from 11 non-RCTs were included. Although age was not reported in all included studies, the participants' age of RCTs and non-RCTs ranged from 34 [46] to 59 [45] and 19 [51] to 62 [52] years, respectively. One RCT [36] and one non-RCT [39] did not report the proportion of gender. For other included RCTs, the proportion between males and females was 101 : 79. For non-RCTs, the proportion between males and females was 62 : 15.

The ASIA level was B, C, or D in RCTs and A, B, C, or D in non-RCTs. The level of injury was cervical (C1-C8) in 80 participants, thoracic in 67, C2-T9 in 46, and above T10 in 30 participants in RCTs. The cervical level of injury was C3 to C7 in 16 participants, thoracic (T3-T12) in 48, lumbar (L1-L5) in 14, and T12-L1 in one participant in non-RCTs.

### 3.2. Interventions

The intervention of RAGT was 3 to 5 sessions per week, 30 min to one hour for 4 to 12 weeks in RCTs. The training protocol of non-RCTs was 2 to 5 sessions, 30 min to 90 min for one week to 90 days.

The apparatus used for RAGT in these studies included Lokomat, hybrid assistive limb (HAL), Indego Exoskeleton, ReWalk, ARKE 2.0, and Ekso GT in which all included RCTs used Lokomat for training.

### 3.3. Risk of Bias of the Included Studies

**Figure 2:**
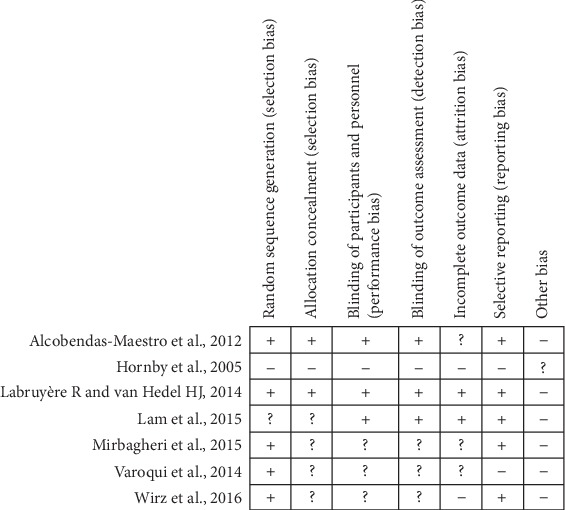
Risk of bias summary for all included RCTs. +: low risk of bias; -: high risk of bias; ?: unclear risk of bias.

Figure 2 summarized the risk of bias judgements related to all RCTs. In all included RCTs, only one study [36] had high risk of bias level. Five studies [37, 38, 44–46] reported randomization. Two studies did not mention randomization [36] or were unclear [47]. Allocation concealment was fulfilled by two studies [44, 45]. Three of the included studies [44, 45, 47] had blinding and the other three studies [37, 38, 46] did not report the methodology of allocation concealment. Two studies [45, 47] did intention to treat analysis and the other three studies [37, 38, 44] were unclear on the information about attrition bias. Two studies [36, 46] had high risk of attrition bias for not reporting their management on the drop-out data.

Table 3 summarized the risk of bias judgements related to non-RCTs. All studies had general to good quality. All non-RCTs recruited representative SCI subjects and no control group. All studies had secure record on training protocol but one study [39] did not. All studies assessed outcomes independently. The duration of three non-RCTs [43, 50, 52] was from 40 min to 2 weeks, most trials with 8 weeks.

### 3.4. Effects of the Interventions

Pain and walking ability were analyzed in RCTs. Spasticity, pain, and walking ability were analyzed in non-RCTs. Summarization on spasticity and TUG in RCTs and FIM-L in non-RCTs were done without meta-analysis due to insufficient data information.

### 3.5. Results of Primary Outcomes: Spasticity

Four RCTs [36, 38, 44, 46] assessed spasticity. However, different muscle groups were assessed in these studies; therefore, the data could not be pooled together. In these studies, all participants' spasticity was mild (MAS 0-2) and none of them changed significantly after RAGT.

**Figure 3:**
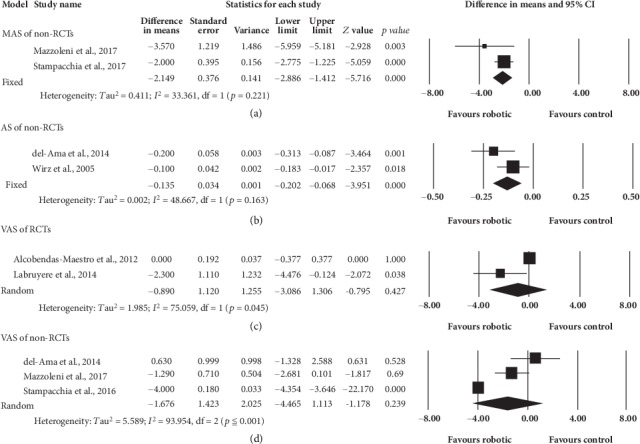
Forest plots of spasticity- (MAS and AS) and pain- (VAS) related variables.

Six eligible non-RCTs were included but only 4 studies' data were retained to pool for analysis. One trial was excluded for analysis because it assessed spasticity of 36 joints together [51]. The other one trial was excluded for analysis because the participants in this trial had no spasticity [52]. Out of the four non-RCTs analyzed, 2 studies [49, 50] use MAS as their outcome measure on 28 participants. The other two studies [42, 43] used AS to assess 23 participants for spasticity (Figure 3). The robotic group showed significant decrease in MAS (95%CI = −2.886 to -1.412, *p* ≤ 0.001) and AS (95%CI = −0.202 to -0.068, *p* ≤ 0.001) measures. The pooled mean difference using MAS and AS (fixed effects model) were -2.149 and -0.135, respectively.

### 3.6. Results of Primary Outcomes: Pain

Two RCTs [44, 45] and 3 non-RCTs [43, 49, 50] were included for analysis. Eighty-four and 31 participants were involved in RCT and non-RCT studies, respectively. Figure 3 showed the results on the analysis of the primary outcomes of pain after RAGT. Although the trend for pain reduction was in favor of robotic group, there was no significant difference between robotic and control group, regardless of RCTs (*p* = 0.427) or non-RCTs (*p* = 0.239). The pooled mean difference (random effects model) of RCTs and non-RCTs were -0.890 and -1.676, respectively. The level of pain ranged from painless [44] to moderate [49, 50] in all included studies.

### 3.7. Results of Secondary Outcomes: LEMS and Walking Ability

#### 3.7.1. LEMS

**Figure 4:**
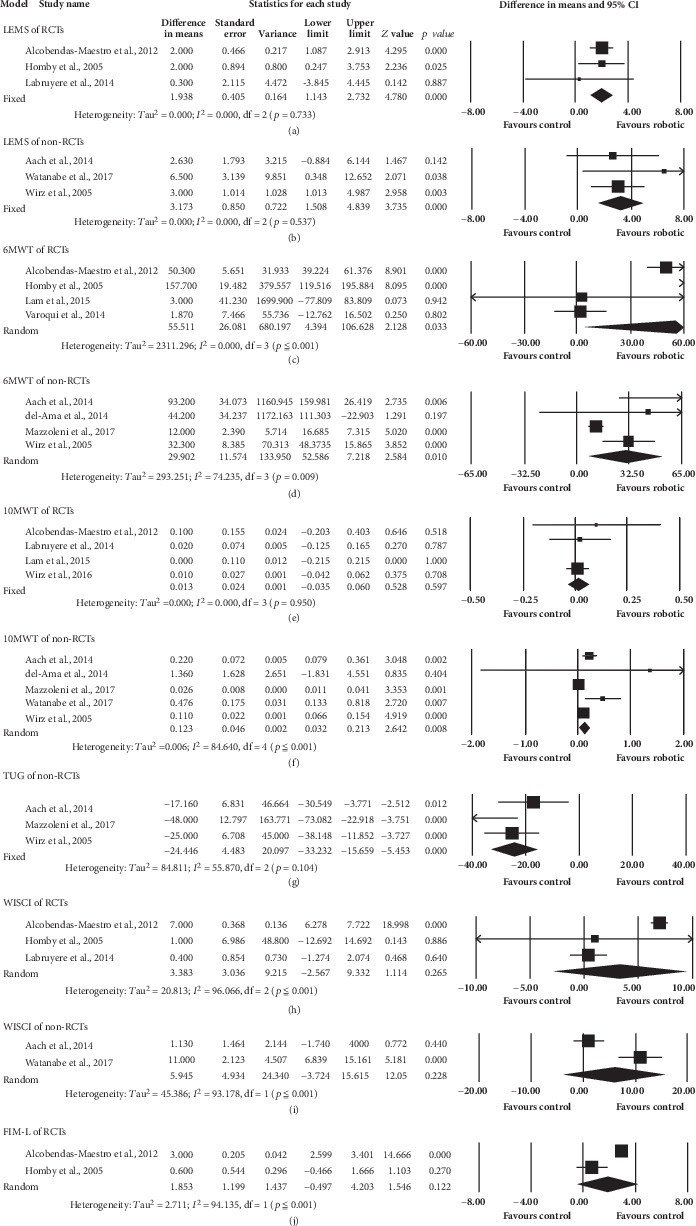
Forest plots of muscle strength of lower limbs (LEMS) and walking ability-related variables (6MWT, 10MWT, TUG, WISCI, and FIM-L).

Three RCTs [36, 44, 45] included 104 participants, and three non-RCTs [42, 52, 53] with 30 participants were pooled for analysis. Significant positive effect in favor of robotic group in both RCTs (95%CI = 1.143 to 2.732, *p* ≤ 0.001) and non-RCTs (95%CI = 1.508 to 4.839, *p* ≤ 0.001) were shown in the results of LEMS. The pooled mean differences (fixed effects model) were 1.938 and 3.173 for RCTs and non-RCTs, respectively (Figure 4).

#### 3.7.2. 6MWT

Four RCTs [36, 38, 44, 47] and 4 non-RCTs [42, 43, 49, 53] assessed 6MWT. A total of 140 and 38 participants were involved in analysis in the RCTs and non-RCTs, respectively. Regardless of RCTs or non-RCTs, walking distance in 6MWT increased significantly in favor of robotic group (RCTs: 95%CI = 4.394 to 106.628, *p* = 0.033; non-RCTs: 95%CI = 7.218 to 52.586, *p* = 0.010). The pooled mean difference (random effects model) of RCTs and non-RCTs were 55.511 m and 29.902 m, respectively (Figure 4).

#### 3.7.3. 10MWT

Five RCTs were included in this analysis, but the data of only four studies were pooled. One study [38] was excluded because no data of control group were provided. Four RCTs [44–47] and 5 non-RCTs [42, 43, 49, 52, 53] were used for subsequent data analysis (Figure 4). In these, 117 and 40 subjects of RCTs and non-RCTs were included, respectively. 10MWT significantly improved in robotic group of non-RCTs (95%CI = 0.032 to 0.213, *p* = 0.008) but not of RCTs (*p* = 0.597). The pooled mean difference (random effects model) for non-RCTs was 0.123 m/s.

#### 3.7.4. TUG

Though one RCT [38] used this outcome measure, there was no sufficient data for analysis. Data from three non-RCTs [42, 49, 53] included 35 participants who were pooled for analysis. The result showed significant improvement in favor of robotic group (95%CI = −33.232 to -15.659, *p* ≤ 0.001). The pooled mean difference (fixed effects model) was -24.446 s (Figure 4).

#### 3.7.5. WISCI

Five RCTs and 3 non-RCTs were included for this analysis, but 2 RCTs and one non-RCT were excluded for insufficient data provided (one RCT [38] and one non-RCT [42]). Data variability of one RCT [46] was too dispersed. Data of three RCTs [36, 44, 45] and 2 non-RCT ones [52, 53] with 104 and 10 participants for RCT and non-RCT, respectively, were finally pooled into analysis, and the results showed no significant difference (*p* = 0.265 for RCTs; *p* = 0.228 for non-RCTs) (Figure 4).

#### 3.7.6. FIM-L

Only 2 RCT studies [36, 44] assessed FIM-L scale. The analysis included 95 subjects. The pooled result showed no significant difference between two groups (*p* = 0.122). The pooled mean difference (random effects model) was 1.853 (Figure 4). For the included non-RCTs, none of them reported FIM-L.

### 3.8. Publication Bias

[Supplementary-material supplementary-material-1] demonstrated the funnel plots of VAS, 6MWT, 10MWT, TUG, WISCI, and LEMS. There were no funnel plots of MAS and AS due to only two studies of each measurement. It seemed a symmetrical funnel plot of VAS, but small study bias was identified in Egger's test (*p* = 0.03289). No small study bias was found in other measurements.

## 4. Discussion

This meta-analysis showed RAGT decreased spasticity and improved walking ability in individuals with SCI. Furthermore, the level of pain showed no change after RAGT.

### 4.1. Spasticity

The current meta-analysis revealed that spasticity decreased after RAGT in non-RCTs. Several possible mechanisms could explain the reduction of spasticity after RAGT. Spasticity is defined as a velocity-dependent increase in tonic stretch reflexes with exaggerated tendon jerks [54]. However, spasticity also involves nonreflex component such as intrinsic muscular properties [54]. Mirbagheri et al. reported that RAGT reduced reflex and intrinsic stiffness of ankle in individuals with SCI [37].

RAGT produces rhythmic movements of lower limbs and provides sensory inputs. Previous studies reported that rhythmic passive exercise could induce spinal circuitry reorganization and decrease spasticity in patients with SCI [27, 55]. Improving spasticity and locomotor function by the activation of spinal locomotor centers might also be influenced by the repetitive elements of the therapeutic program [56]. RAGT is a type of repetitive functional task training. These above mechanisms might possibly explain the finding that RAGT reduces spasticity.

The reasons that the RCTs did not show significant reduction in spasticity in the RAGT group might be due to the floor effect [44, 46] (MAS 0 to 1) and the measurements done on different joints [38, 44, 46]. In the included studies, the subjects' initial spasticity level was not high enough to show change after RAGT. It is suggested that subjects with more severe spasticity could be recruited for further investigation of RAGT.

### 4.2. Pain

Pain and spasticity are intricate consequences of spinal cord injury [57]. Researchers suggested that pain and spasticity are closely linked [57]. In addition, pain and spasticity might share similar pathophysiological mechanisms [5]. Hence, it is reasonable to expect a reduction of pain with spasticity reduction after RAGT. However, the result of this meta-analysis did not show significant decrease of pain accompanying reduced spasticity following RAGT. This might be that the pain suffered by the participants in these included trials was not mainly from spasticity. Some other potential sources, such as muscle soreness due to excess exercise, joint pain due to malposture, or poor biomechanics, might be the cause of pain. One should also note that the neuropathic pain, more than 50% prevalence in spinal cord injured persons [58], was not reported in the included studies. Therefore, they lacked source of data for meta-analysis. It is suggested to be investigated in future studies.

Although, the included RCTs and non-RCTs did not show significant change in pain, the trend favored RAGT group. Past studies revealed that physical activities could relieve musculoskeletal and neuropathic pain [59, 60]. However, participants in the current meta-analysis did not subjectively feel significant alteration in pain level with VAS assessment. The baseline floor effect of mild [44] to moderate [50] intensity of pain felt by the participants might account for the nonsignificant result. Future studies that include participants with higher level of pain at baseline are suggested.

### 4.3. LEMS

This study showed that LEMS significantly improved after RAGT. As discussed previously, rhythmic muscle activations could be detected during RAGT. In addition, weight bearing may be an important factor. RAGT provides support which allows users to load their weight on lower limbs during training. Lower limbs weight bearing and the enhancement of muscle activation may contribute to the improvement of LEMS.

Decreasing guiding force as RAGT progresses might increase the muscle strength of lower extremities. Subjects needed greater engagement to activate muscles and participate in the training program. Since the guiding force has not been quantified in the included studies, investigation of the relationship between guiding force and the improvement of LEMS is suggested in future studies.

### 4.4. 6MWT

This meta-analysis showed that 6MWT increased significantly in favor of the RAGT group. 6MWT is an indicator of endurance. Clinically meaningful change (CMC) of 6MWT was 19-22 m in healthy older adults [61]. The 95% CI of the current meta-analysis includes the range 4.394-106.628 m for included RCTs and 7.218-52.586 m for included non-RCTs. Hence, RAGT can be clinically practical for endurance training.

In physiological point of view, endurance could be improved by multiple sessions of submaximal voluntary exercises [62]. RAGT, due to its lack of active participation from the users during training, was doubted to increase cardiopulmonary fitness in subjects [63]. However, increased 6MWT in this meta-analysis indicates it could improve endurance without emphasizing voluntary muscle contraction. According to Mazzoleni et al. [64], bilateral muscular activity increased after RAGT in people with SCI. Thus, the activation of muscles might increase the challenge to cardiopulmonary systems and, thus, increase the endurance of participants with SCI.

### 4.5. Walking Speed (10MWT and TUG)

TUG is commonly used to assess functional mobility. It was correlated with muscle strength of the lower extremities and gait speed [18, 65]. The current meta-analysis showed that both parameters improved after RAGT in SCI. The results of 10MWT also supported that RAGT increased walking speed in SCI. The CMC of 10MWT was 0.04-0.06 m/s in healthy older adults [61]. The pooled mean difference (0.123 m/s) was above CMC in included non-RCTs in this meta-analysis.

Kim et al. [66] reported that muscle strength of the lower extremities was correlated with walking speed in chronic incomplete SCI. As shown with the result of LEMS's improvement, lower extremity strength might be the cause of improved walking speed.

Another explanation for improved walking speed was the strengthening of central pattern generator (CPG). Previous studies supported that RAGT, which involved rhythmic activations of lower extremities, could strengthen CPG [67]. CPG is an essential neural mechanism of walking. Enhanced CPG would lead to increase walking ability. The other explanation was the reduction of spasticity. Spasticity could increase the resistance of movement and interfere with gait. As shown in the above result, spasticity reduced after RAGT and, thus, resulted in less resistance during walking.

### 4.6. WISCI and FIM-L

WISCI and FIM-L showed no significant difference after RAGT in this meta-analysis. The reason might be that studies investigated that WISCI and FIM-L were few in the current meta-analysis. However, the CMC for the WISCI was 1 point [68]. The pooled mean differences of included studies all exceed CMC (3.383 for RCTs and 5.945 for non-RCTs).

FIM was graded according to the assistance required by a subject. People with SCI might experience fear of falling that impeded transferring the improved walking abilities to functional tasks after RAGT.

### 4.7. Study Limitations

The first limitation of this current review is no classification of subgroups according to the level and severity of SCI. More trials and subjects are needed for subgroup analysis. The second limitation is the risk of bias exists in all studies. Compared with the non-RCTs, the number of RCTs studies is relatively few. The third limitation is that the training protocol used in each study is not identical. Further reviews are suggested to compare effects of different protocols with increased trials.

## 5. Conclusions

This meta-analysis concluded that RAGT had positive effects in improvements of spasticity and walking ability. In RCTs, walking distance and muscle strength of lower limbs improved after RAGT. RAGT can be applied in individuals with SCI without increasing pain.

## Figures and Tables

**Table 1:** Characteristics of included RCTs.

Study	Research design	Participants	Intervention	Outcome measures
Alcobendas-Maestro et al., [44]	RCT	*n* = 75 (37 in the Lokomat training group, 38 in the conventional overground group) ASIA C or D Level of injury: C2 to T12	40 sessions over 8 weeks, 1 hour (1) Lokomat group: 30 min with the Lokomat in each session+30 min standard physical treatment (2) Overground group: one hour standard physical treatment	AS VAS 6MWT, 10MWT WISCI II FIM-L, LEMS

Hornby et al., [36]	RCT	*n* = 30 ASIA B, C, D Level of injury: above T10	(1) Robotic-assisted BWSTT (2) Therapist-assisted BWSTT (3) Overground ambulation with a mobile suspension system three 30-minute sessions per week, 8 weeks	AS SCATS 6MWT, 10MWT WISCI FIM-L LEMS, TUG

Labruyère and van Hedel, [45]	RCT cross over	*n* = 9 ASIA D Level of injury: C4 to T11	(1) Group 1: 16 sessions of RAGT (Lokomat) followed by 16 sessions of strength training (2) Group 2: the same intervention in reversed order	VAS, 10MWT, WISCI LEMS, UEMS, FET, PCI, gait symmetry, BBS Body sway FES-I, SCIM

Lam et al., [47]	RCT	*n* = 15 ASIA C or D Lesion level below thoracic 11 or lower motoneuron injury was excluded	(1) Lokomat-resisted BWSTT (Loko-R) (2) Lokomat-assisted BWSTT (control) 45 min, 3 times/week for 3 mo.	Reports of pain 10MWT 6MWT SCI-FAP

Mirbagheri et al., [37]	RCT	*n* = 46 ASIA C or D Level of injury: C2 to T9	(1) RAGT group: 3 times a week over four weeks, one hour/session (2) Control group: no intervention	MAS Intrinsic stiffness K Reflex stiffness G

Varoqui et al., [38]	RCT	*n* = 30 ASIA C or D Level of injury: above T10	(1) Lokomat group: 3 times a week over four weeks, one hour/session (2) Control group	MAS 10MWT 6MWT TUG Ankle kinematic and kinetic assessments

Wirz et al., [46]	RCT	*n* = 21 ASIA B or C Level of injury: C4 to T12	(1) Intervention group: 50 min/training (2) Control group: 25 min/training Lokomat, 3-5 days/week, 8 weeks	MAS SCIM III WISCI II Penn, GICS

BWSTT: body-weight supported treadmill training; AS: Ashworth scale; MAS: modified Ashworth scale; 10MWT: 10-meter walking test; 6MWT: 6-minute walk test; WISCI: Walking Index for Spinal Cord Injury; FIM-L: Functional Independence Measure-Locomotor section; LEMS: lower extremity motor score; SCIM: Spinal Cord Independence Measure; Penn: Modified Penn Spasm Frequency Scale; TUG: timed up and go test; FET: Figure Eight Test; PCI: Physiological Cost Index; BBS: Berg balance scale; FES-I: falls efficacy scale-international version; UEMS: upper extremity motor score; SCI-FAP: Spinal Cord Injury-Functional Ambulation Profile; SCATS: Spinal Cord Assessment Tools for Spasticity; GICS: Global Impression of Change Scale.

**Table 2:** Characteristics of included non-RCTs.

Study	Research design	Participants	Intervention	Outcome measures
Aach et al., [53]	Single case experimental A-B (pre-post) design	*n* = 8 ASIA A Level of injury: T8 to L2	HAL 5 times per week, 90 days, mean number of sessions of 51.75 ± 5.6	6MWT, 10MWT TUG WISCI II LEMS Muscle volume

Del-Ama et al., [43]	Pilot study	*n* = 3 ASIA A and D Level of injury: L1, L4, L5	Kinesis system The first week intervention, the second week no intervention	VAS AS 10MWT 6MWT Penn MMT

Ekelem and Goldfarb, [39]	Case report	*n* = 2 ASIA B Level of injury: T4, T11	Indego exoskeleton practice < 4 hr per day	MAS

Esquenazi et al., [40]	Prospective, single-intervention	*n* = 12 ASIA B Level of injury: T3-T12	ReWalk Up to 24 sessions of 60 to 90 min duration over approximately 8 weeks (target was three times per week)	Pain, fatigue (VAS) AS HR, BP Skin integrity

Ikumi et al., [51]	Case report	*n* = 1 ASIA A Level of injury: C4	HAL 60 min, 2 times per week for 5 weeks in addition to standard physical and occupational therapy	MAS Walking time and distance

Lemaire et al., [48]	Case report	*n* = 2 ASIA B Level of injury: T6, T12	ARKE 2.0 LEPE 12 half-hour training sessions, four or more weeks	Ten-point scale: pain, fatigue Number of steps Distance travelled Standing duration Walking duration Number of partial steps

Manella et al., [41]	Case report	*n* = 1 ASIA A Level of injury: T7	Lokomat 40 mins, 3 times per week, 12 weeks	AS Pendulum test of quadriceps spasticity Spasm frequency and severity ASIA sensory scores LEMS FIM

Mazzoleni et al., [49]	Single group	*n* = 7 ASIA A Level of injury: T4-T12	20 sessions, 3 sessions/week, FES-cycling system (Pegaso) followed by 20 sessions, 3 sessions/wk, overground robotic exoskeleton (Ekso GT)	MAS PSFS SCIM Spasticity and pain through a 0-10 points NRS ISCI 6MWT, 10MWT TUG

Stampacchia et al., [50]	Single group	*n* = 21 ASIA A, B, D Level of injury: C7, L1-L2, dorsal	Ekso GT 40 min	Pain and spasticity (0-10 points scale) MAS PSFS PGIC

Watanabe et al., [52]	Case report	*n* = 2 ASIA C, D Level of injury: T8-T10, T12-L1	HAL 3-4 times per week, for a total of 8 sessions, in addition to conventional physical therapy, 20-30 min/session	MAS LEMS WISCI II FIM CGS Stride, cadence Right and left leg swing time Hip and knee joint angle BI, mRS and adverse effects

Wirz et al., [42]	Single group	*n* = 20 AISA = C or D Level of injury: L1 or higher	Lokomat (DGO) 8 weeks, 3 to 5 sessions each week, 45 min	Primary: WISCI II, 10MWT, 6MWT, TUG Secondary: at 1 center, *n* = 10 LEMS, AS, SCATS

AS: Ashworth scale; MAS: modified Ashworth scale; 10MWT: 10-meter walking test; 6MWT: 6-minute walk test; WISCI: Walking Index for Spinal Cord Injury; FIM: Functional Independence Measure; LEMS: lower extremity motor score; HR: heart rate; BP: blood pressure; SCIM: Spinal Cord Independence Measure; Penn: Modified Penn Spasm Frequency Scale; TUG: timed up and go test; SCATS: Spinal Cord Assessment Tools for Spasticity; PSFS: Penn Spasm Frequency Scale; ISCI: International Spinal Cord Injury Pain Data Set; PGIC: patient's global impression of change; CGS: comfortable gait speed; BI: Barthel index; mRS: modified Rankin Scale.

**Table 3:** Assessment of study quality with Newcastle-Ottawa scale.

		Selection				Comparability		Outcome			
Study ID	Year	S1	S2	S3	S4	C1	C2	O1	O2	O3	No. of star
Aach et al.	2014	★		★	★			★	★	★	6
del-Ama et al.	2014	★		★	★			★		★	5
Ekelem and Goldfarb	2018	★			★			★			3
Esquenazi et al.	2012	★		★	★			★	★	★	6
Ikumi et al.	2017	★		★	★			★	★	★	6
Lemaire et al.	2017	★		★	★			★	★	★	6
Manella et al.	2010	★		★	★			★	★	★	6
Mazzoleni	2017	★		★	★			★	★	★	6
Stampacchia	2016	★		★	★			★		★	4
Watanabe	2017	★		★	★			★		★	4
Wirz et al.	2005	★		★	★			★	★	★	6

## Abbreviations

SCI: Spinal cord injury
RAGT: Robot-assisted gait training.

## Supplementary Materials

Supplementary Materials Appendix 1: searching keywords. Appendix 2: funnel plots and Egger's test of (a) VAS for non RCTs, (b) 6MWT for 3 RCTs, (c) 6MWT for non RCTs, (d) 10MWT for RCTs, (e) 10MWT for non RCTs, 4 (f) TUG for non RCTs, (g) WISCI for RCTs, (h) LEMS for RCTs, and (i) LEMS for non 5 RCTs.
