# Supplementary material for: Effects of Robot-Assisted Gait Training in Individuals with Spinal Cord Injury: A Meta-analysis
Source: Biomed Res Int. 2020 Mar 21;2020:2102785. doi: 10.1155/2020/2102785 (PMC7115057; doi:10.1155/2020/2102785)
Supplement: Supplementary Materials — Appendix 1: searching keywords. Appendix 2: funnel plots and Egger's test of (a) VAS for non RCTs, (b) 6MWT for 3 RCTs, (c) 6MWT for non RCTs, (d) 10MWT for RCTs, (e) 10MWT for non RCTs, 4 (f) TUG for non RCTs, (g) WISCI for RCTs, (h) LEMS for RCTs, and (i) LEMS for non 5 RCTs. [file 2102785.f1.pdf]

Appendix 1 Searching keywords

SCI related

1. Spinal cord injury
2. SCI
3. Spinal cord ischemia
4. Spinal cord vascular diseases
5. Spinal cord neoplasm
6. Spinal cord disease
7. Spinal cord compression
8. Spinal cord laceration
9. Myelopathy
10. Spinal tumor
11. Spinal damage
12. Spinal trauma
13. Spinal fracture

Robot related

14. Robot
15. Robot assisted gait training

16. Exoskeleton
17. Body weight supported treadmill training
18. BWSTT
19. Locomat
20. Lokomat
21. Orthotic
22. Orthosis

Spasticity related

23. Spasticity
24. Hypertone
25. Spasm
26. Clonus

Pain related

27. Pain
28. Chronic pain
29. Musculoskeletal pain
30. Neurological pain
31. 1 or 2 or 3 or 4 or 5 or 6 or 7 or 8 or 9 or 10 or 11 or 12 or 13

32. 14 or 15 or 16 or 17 or 18 or 19 or 20 or 21 or 22

33. 23 or 24 or 25 or 26

34. 27 or 28 or 29 or 30

35. 31 and 32 and 33 and 34

Appendix 2 Funnel plots and Egger's test of (A) VAS for non RCTs, (B) 6MWT for RCTs, (C) 6MWT for non RCTs, (D) 10MWT for RCTs, (E) 10MWT for non RCTs, (F) TUG for non RCTs, (G) WISCI for RCTs, (H) LEMS for RCTs, (I) LEMS for non RCTs,

A. VAS for non RCTs

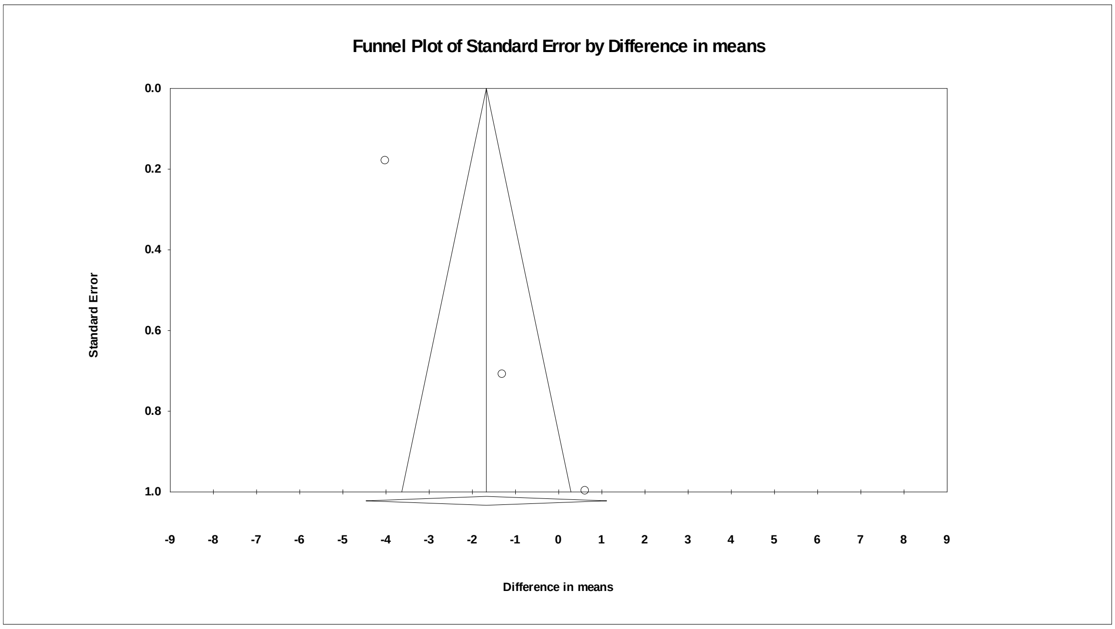

Egger's regression intercept

Intercept	5.41829
Standard error	0.28015
95% lower limit (2-tailed)	1.85863
95% upper limit (2-tailed)	8.97796
t-value	19.34057
df	1.00000
P-value (1-tailed)	0.01644
P-value (2-tailed)	0.03289

B. 6MWT for RCTs

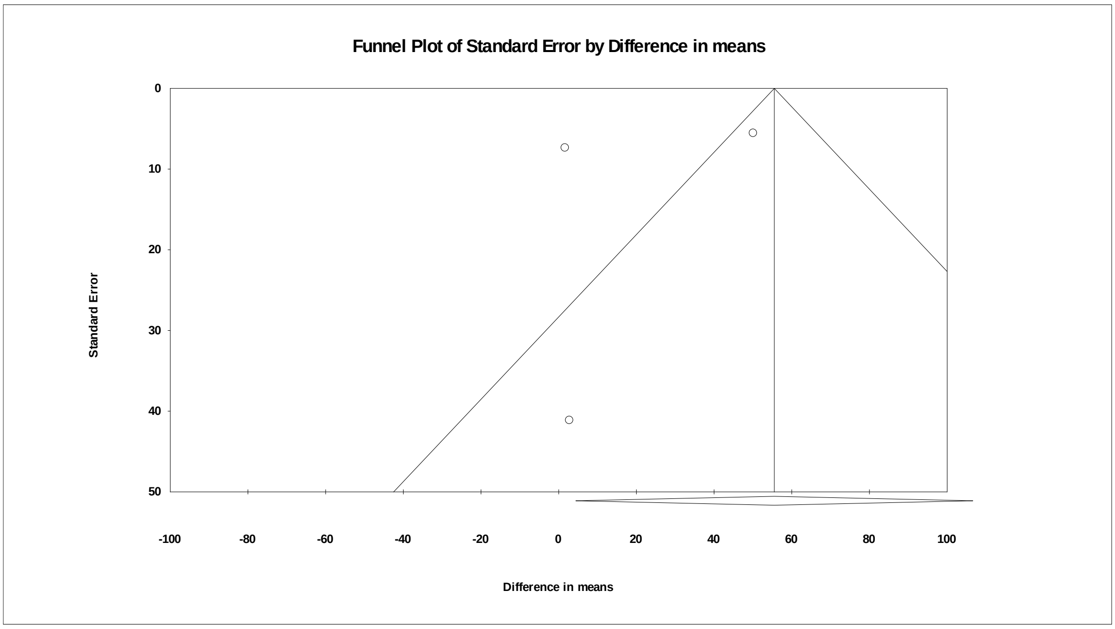

Egger's regression intercept

Intercept	2.07918
Standard error	5.16789
95% lower limit (2-tailed)	-20.15645
95% upper limit (2-tailed)	24.31481
t-value	0.40233
df	2.00000
P-value (1-tailed)	0.36318
P-value (2-tailed)	0.72637

C. 6MWT for non RCTs

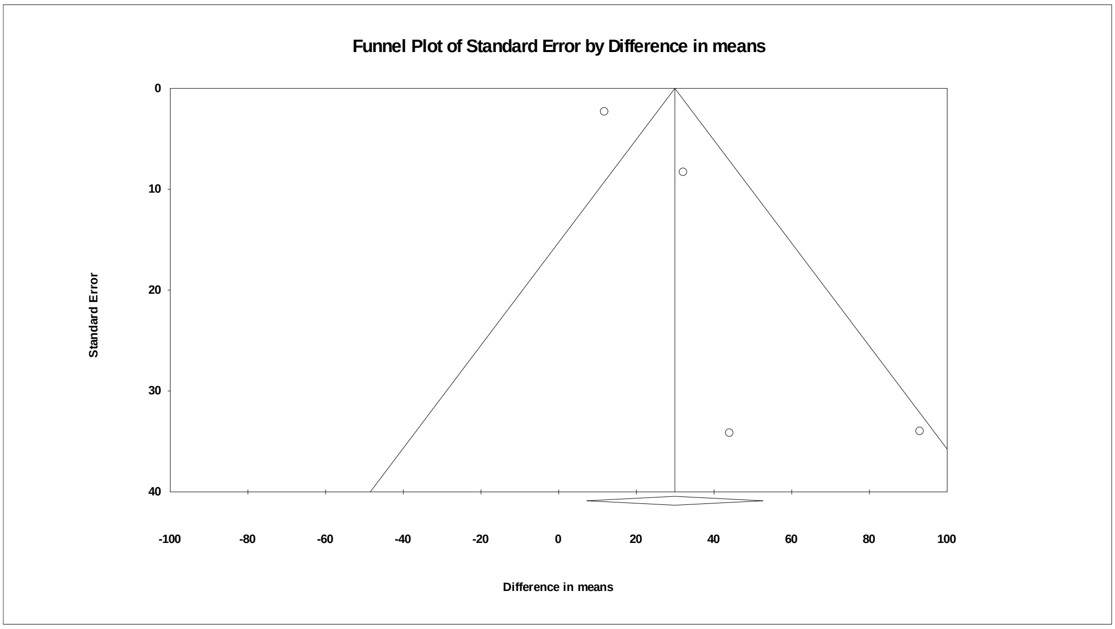

Egger's regression intercept

Intercept	2.12231
Standard error	0.68563
95% lower limit (2-tailed)	-0.82771
95% upper limit (2-tailed)	5.07233
t-value	3.09542
df	2.00000
P-value (1-tailed)	0.04522
P-value (2-tailed)	0.09043

D. 10MWT for RCTs

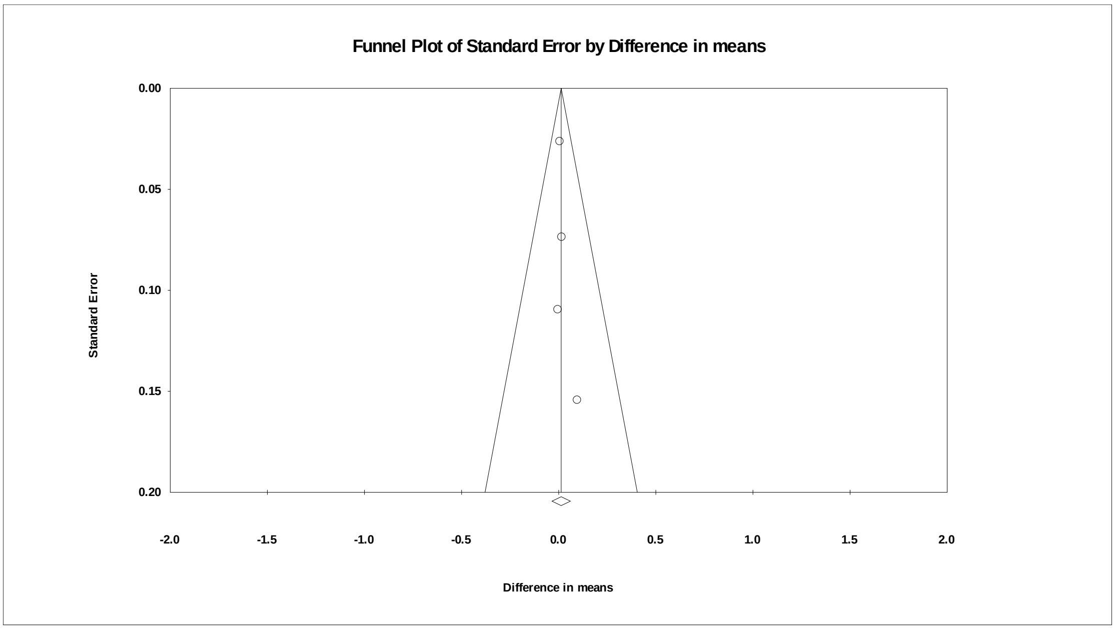

Egger's regression intercept

Intercept	0.31210
Standard error	0.27530
95% lower limit (2-tailed)	-0.87242
95% upper limit (2-tailed)	1.49663
t-value	1.13369
df	2.00000
P-value (1-tailed)	0.18726
P-value (2-tailed)	0.37453

E. 10MWT for non RCTs

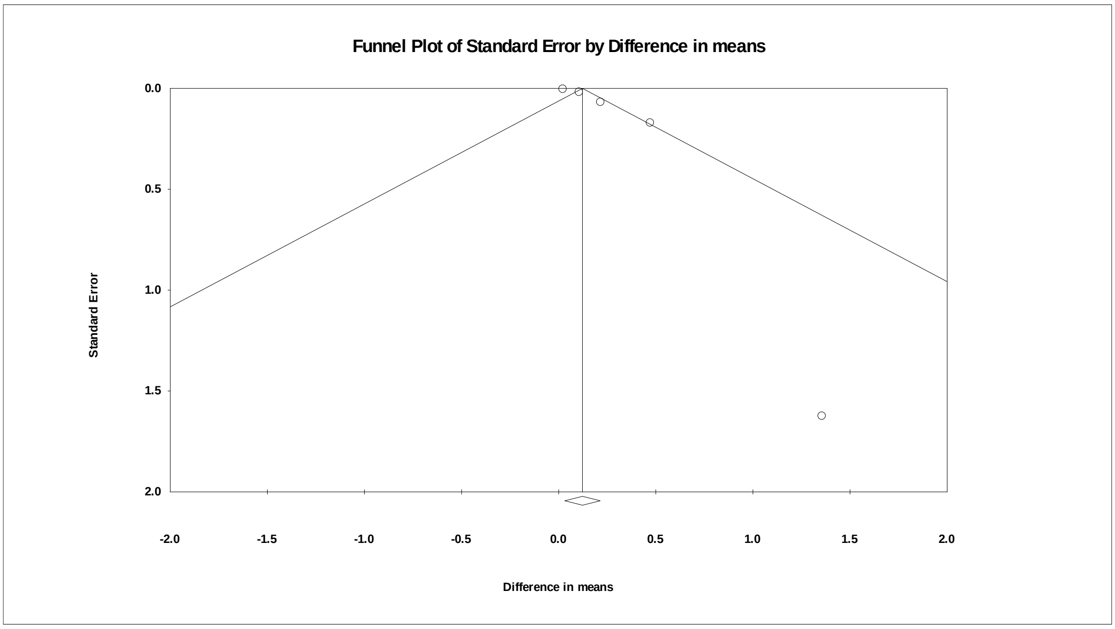

Egger's regression intercept

Intercept	2.51721
Standard error	0.88036
95% lower limit (2-tailed)	-0.28449
95% upper limit (2-tailed)	5.31891
t-value	2.85929
df	3.00000
P-value (1-tailed)	0.03231
P-value (2-tailed)	0.06461

F. TUG for non RCTs

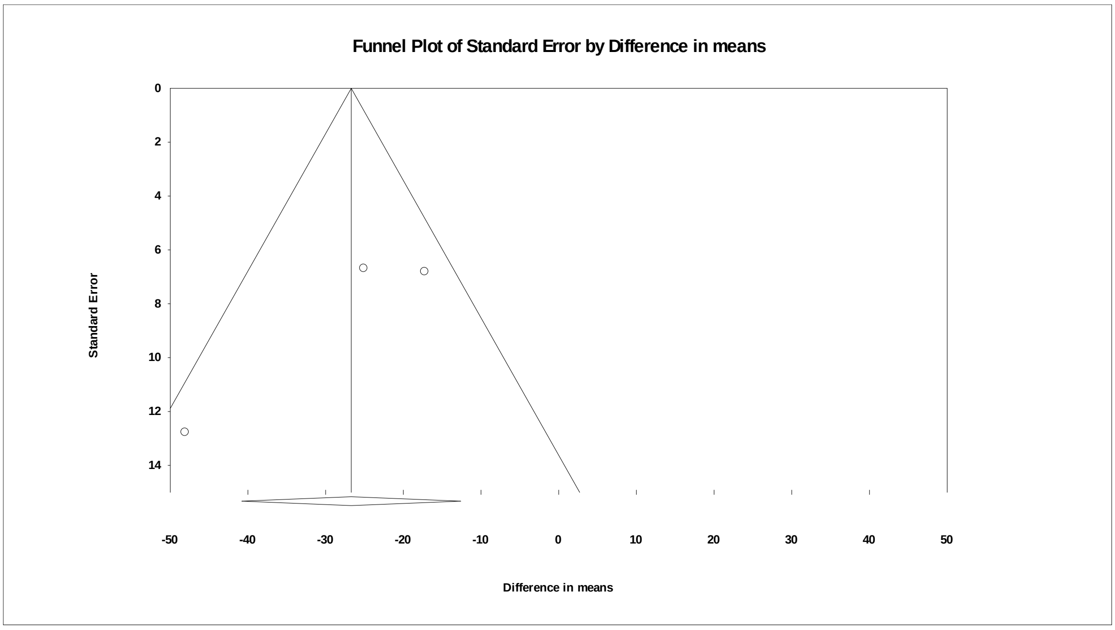

Egger's regression intercept

Intercept	-4.39574
Standard error	1.98369
95% lower limit (2-tailed)	-29.60090
95% upper limit (2-tailed)	20.80943
t-value	2.21594
df	1.00000
P-value (1-tailed)	0.13494
P-value (2-tailed)	0.26987

G. WISCI for RCTs

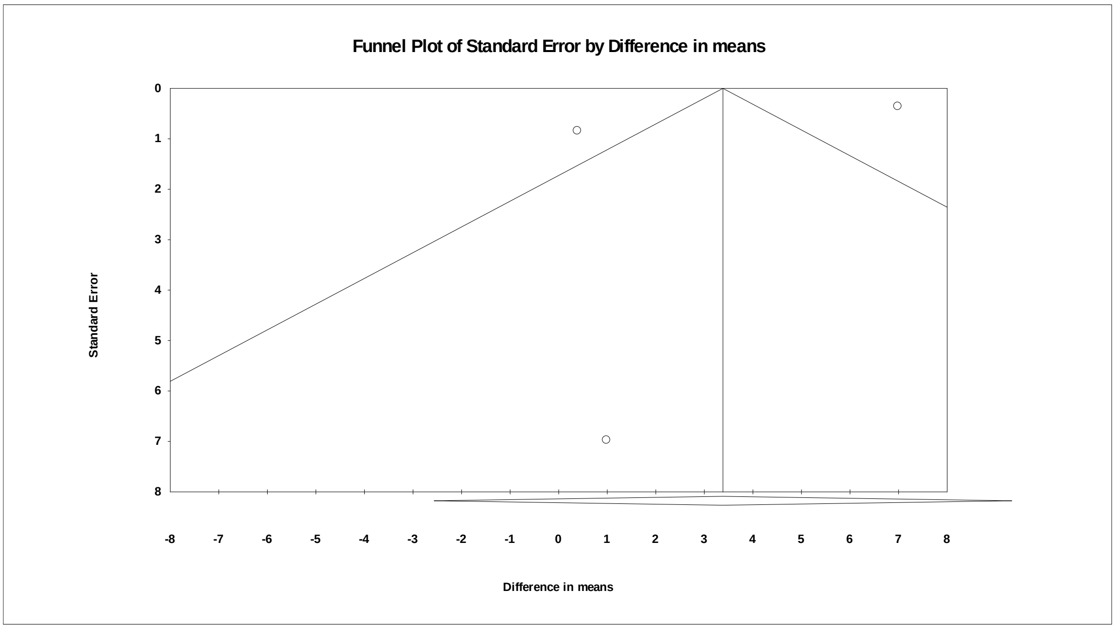

Egger's regression intercept

Intercept	-3.80748
Standard error	5.46069
95% lower limit (2-tailed)	-73.19217
95% upper limit (2-tailed)	65.57720
t-value	0.69725
df	1.00000
P-value (1-tailed)	0.30619
P-value (2-tailed)	0.61238

H. LEMS for RCTs

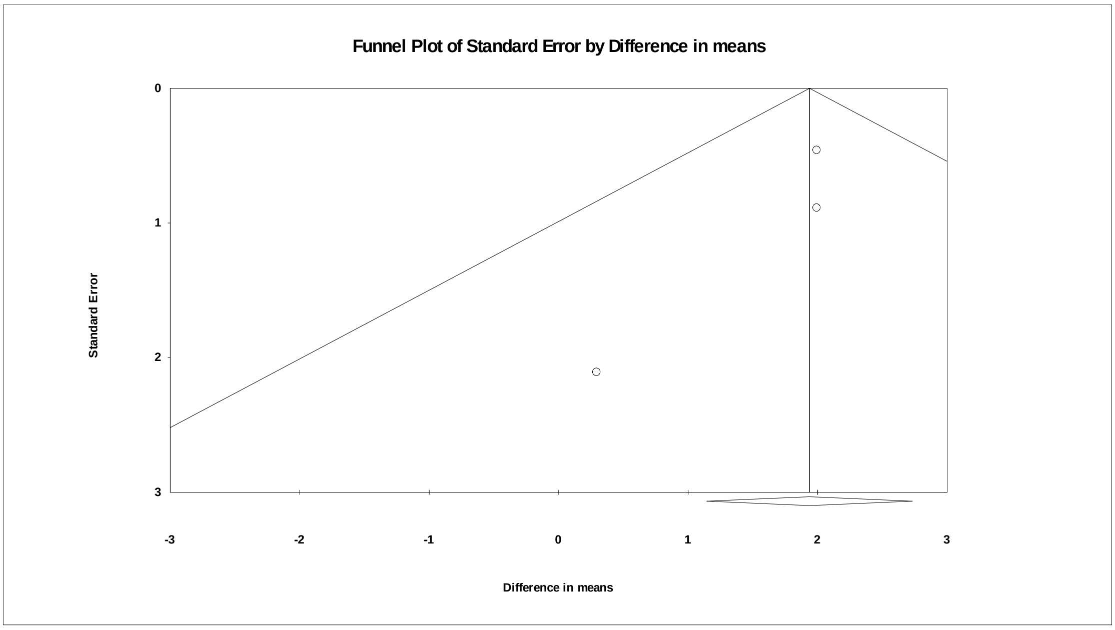

Egger's regression intercept

Intercept	-0.81089
Standard error	0.47697
95% lower limit (2-tailed)	-6.87132
95% upper limit (2-tailed)	5.24954
t-value	1.70011
df	1.00000
P-value (1-tailed)	0.16924
P-value (2-tailed)	0.33849

I. LEMS for non RCTs

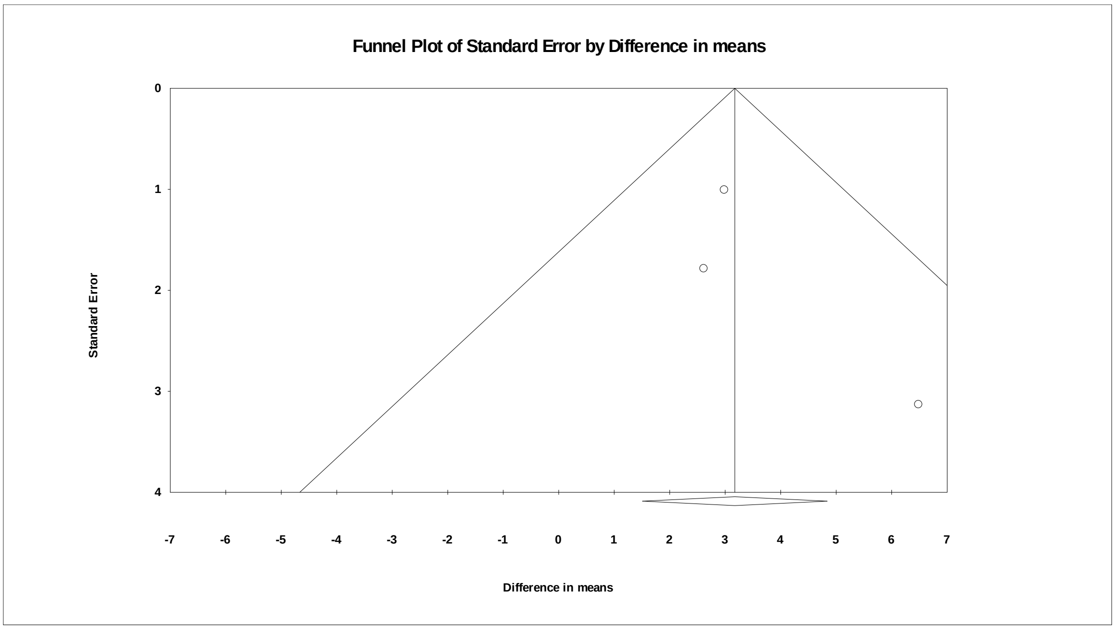

Egger's regression intercept

Intercept	1.18206
Standard error	1.05544
95% lower limit (2-tailed)	-12.22859
95% upper limit (2-tailed)	14.59270
t-value	1.11996
df	1.00000
P-value (1-tailed)	0.23201
P-value (2-tailed)	0.46401
